# Dietary Acid Load Is Not Associated with Serum Testosterone in Men: Insights from the NHANES

**DOI:** 10.3390/nu15133075

**Published:** 2023-07-07

**Authors:** Maximilian Andreas Storz, Alvaro Luis Ronco

**Affiliations:** 1 Department of Internal Medicine II, Centre for Complementary Medicine, Freiburg University Hospital, Faculty of Medicine, University of Freiburg, 79106 Freiburg, Germany; 2 Unit of Oncology and Radiotherapy, Pereira Rossell Women’s Hospital, Bvard. Artigas 1590, Montevideo 11600, Uruguay; alv.ronco58@gmail.com

**Keywords:** dietary acid load, potential renal acid load, net endogenous acid production, sex hormones, testosterone, nutritional epidemiology, men’s health

## Abstract

The dietary acid load (DAL) is a novel marker of overall diet quality, which has been associated with overweight, type 2 diabetes and altered glucocorticoid secretion. A potential association with sex hormones is thus not inconceivable. We investigated whether DAL was associated with serum total testosterone concentrations of men in the National Health and Nutrition Examination Survey. The DAL scores, including the potential renal acid load (PRAL) and net endogenous acid production (NEAP), were estimated and compared between participants with low and normal testosterone levels. The investigated sample encompassed *n* = 377 males with a mean age of 49.50 years. Approximately 73% of the sample were of Non-Hispanic White origin. None of the examined DAL scores showed significant associations with serum testosterone levels. We observed no significant differences in the crude DAL scores between individuals with low testosterone levels and individuals with normal testosterone levels. Multivariate regression models adjusting for covariates confirmed the lack of associations between the PRAL and serum testosterone. Our results are of particular importance for those individuals who wish to lower their DAL in light of the presumable health effects of a more alkaline diet. Our data suggest that diet modifications toward a lower intake of animal protein and refined grains (which consecutively translates into a lower DAL) may not negatively affect men’s testosterone levels.

## 1. Introduction

Testosterone levels in the United States male population have declined within the last few decades [1,2]. Modifiable lifestyle factors, including a Western-style diet abundant in processed meats and saturated fat, have been discussed as potential contributors to this phenomenon [3,4,5,6].

Specific dietary patterns and nutrients have been reported to influence sex steroid hormone levels in both observational and clinical studies [7]. Diet has the potential to alter sex hormone production, metabolism, excretion, and bioavailability [8]. Testosterone and estradiol are the major sex steroids in the human body [7], and they play important roles in the regulation of various processes in the cardiovascular, immune, muscular and neural systems [9]. Testosterone further acts as an anabolic hormone, contributing to muscle mass, penile enlargement and libido as well as spermatogenesis in men [10].

More than 30 years ago, Adlercreutz summarized his research findings suggesting that a Western diet elevates the plasma levels of sex hormones and decreases the serum sex hormone-binding globulin concentrations, thereby increasing the bioavailability of these steroids [8]. Since then, various studies examined the potential associations between dietary patterns (or specific foods) and sex steroid hormones in men [7,11,12]. Notably, said studies showed mixed and partly inhomogeneous results.

Zhang et al. reported that men adhering to a more pro-inflammatory diet (as measured by the Dietary Inflammatory Index (DII)) appeared to have a higher risk of testosterone deficiency [13]. The DII is an epidemiological tool used to characterize the inflammatory potential of an individual diet [14], and it was also used in a study by Qin et al., who reported a similar association between a pro-inflammatory diet and lower total testosterone levels in male adolescents [15]. On the other hand, the Healthy Eating Index (HEI)—a scoring metric that can be used to determine overall diet quality—was not associated with total or free testosterone in a study by Chen et al. [7,16]. A lack of associations between the Plant-based Diet Index (PDI) (and the Healthful Plant-based Diet Index (hPDI), respectively) and testosterone levels were reported by Lu et al. in young healthy Chinese men [17] and by Kuchakulla et al. in U.S. males [12].

In light of the aforementioned studies, we hypothesized that general eating patterns may influence men’s health via changes in sex steroid hormones. One potential overall diet quality index that has not been investigated in this context is the dietary acid load (DAL). The DAL measures the diet’s impact on the acid–base balance in humans [18]. Meat (e.g., beef, poultry, fish), dairy, and grains confer higher acid loads, whereas fruits, vegetables, and legumes tend to be neutral or have an overall negative impact on the DAL due to their high content of alkali precursors [19].

A high DAL may negatively impact cardiometabolic health and could thereby (hypothetically) impact testosterone levels in men [20,21]. Although DAL is an emerging overall dietary quality marker of current clinical and epidemiological interest [22], its potential association with sex hormones remains largely unexplored. Thus, we aimed to investigate whether the DAL was associated with serum total testosterone concentrations of men in the US-based National Health and Nutrition Examination Survey (NHANES).

## 2. Materials and Methods

### 2.1. Study Population and Design

Our analysis is based on cross-sectional aggregated population-based data from the NHANES [23,24]. The NHANES is an ongoing program of studies by the Centers for Disease Control and Prevention (CDC) that was designed to assess the health and nutritional status of the non-institutionalized U.S. population. Since the 1960s, the NHANES has been conducted as a series of surveys focusing on different population groups and various health topics. The NHANES uses a complex, multistage, stratified, clustered and probability sampling design that allows for nationally representative health status assessments. The sample for the survey is representative of the non-institutionalized U.S. population of all ages. The NHANES examines a sample of approximately 5000 individuals located across the U.S. per annum. One of the major aims is to identify the health-care needs of the United States population, which supports government agencies and other institutions in establishing policies and health promotion programs to improving population health [24]. The NHANES, its history, its background and its program structure have been described elsewhere in great detail [23,24]. Household questionnaires, interviews by phone, as well as clinical examinations conducted by health-care professionals and trained personnel were utilized to collect data [25]. All study participants gave written and oral consent to participate the study, which was approved by the National Center for Health Statistics (NCHS) [26].

### 2.2. Assessment of Testosterone, Estradiol and Sex Hormone-Binding Globulin

The sex steroid hormone levels, including the total testosterone (in ng/dL), estradiol (in pg/mL) and sex hormone-binding globulin (SHBG, nmol/L), were obtained from the 2015–2016 laboratory data module [27]. The laboratory methodology has been described elsewhere in detail [27]. In brief, the total testosterone and estradiol in the serum were simultaneously measured using isotope dilution liquid chromatography tandem mass spectrometry (ID-LC-MS/MS) method for routine analyses developed by the CDC. The aforementioned method was created for high sample throughput and showed sufficient precision and high accuracy for a long time. The method was certified by the CDC Hormone Standardization Program and is traceable to certified reference materials obtained from the Australian National Measurement Institute and the National Metrology Institute of Japan [27,28]. The SHBG measurement was based on the reaction of SHBG with immuno-antibodies and chemo-luminescence measurements of the reaction products that occurs after two incubation periods and subjecting to a magnetic field [27]. Additional procedure details may be obtained from the official NHANES laboratory procedure manual for estradiol and testosterone [29] as well as for SHBG [30]. The lower limits of detection for testosterone, estradiol and SHBG were as follows: 0.75 ng/mL, 2.994 pg/mL, and 0.800 nmol/L, respectively. Although of potentially limited value in men, we finally calculated the free androgen index (FAI) as described earlier by Kapoor et al. [31,32].

### 2.3. Dietary Acid Load Markers and Nutrient Intake

The DAL calculation methods have been described elsewhere in great detail [33]. Formulas by Remer and Manz [34,35] and Frassetto and colleagues were used to estimate the potential renal acid load (PRAL) and net endogenous acid production (NEAP) [36]. Based on Remer’s formula, we estimated the PRAL_R_ (in mEq/d) as follows:

PRAL_R_ (mEq/day) = (0.49 × total protein intake (g/d)) + (0.037 × phosphorus intake (mg/d)) − (0.021 × potassium intake (mg/d)) − (0.026 × magnesium intake (mg/d)) − (0.013 × calcium intake (mg/d))

The NEAP was estimated based on Remer’s formula (NEAP_R_) [34], and based on Frassetto’s formula (NEAP_F_) [36]. The latter considers the daily potassium intake and protein intake:

NEAP_F_ = (mEq/d) = (54.4 × protein (g/d)/potassium (mEq/d)) − 10.2

The formula for the NEAP by Remer and Manz (NEAP_R_) considers the PRAL_R_ score and anthropometry-based estimates of organic acid excretion, whereby the OAest was estimated as follows:

Individual body surface area × 41/1.73

We estimated the NEAP_R_ (in mEq/d) as follows:

Estimated NEAP_R_ (mEq/d) = PRAL (mEq/d) + OAest (mEq/d)

The micro- and macronutrient intake estimates required for the DAL calculations were drawn from the NHANES dietary interview module, aiming to derive detailed dietary intake information from the NHANES participants [37]. The dietary interview component, called What We Eat in America (WWEIA), which is conducted as a partnership between the U.S. Department of Health and Human Services (DHHS) and the U.S. Department of Agriculture (USDA), has been described elsewhere in detail [38,39]. The nutrient and total energy intake for all the participants was estimated based on a computerized 24 h dietary recall method. The dietary recall validity, its clinical applicability and its methodology have been described previously [38,39,40,41].

### 2.4. Other Potential Confounders and Covariates

The covariates in this study included demographic data (age, gender, race/ethnicity, marital status, education level), anthropometric data (body mass index (BMI)) and various lifestyle factors (including physical activity, alcohol intake, smoking status, and hours of sleep per night). Age was treated as a continuous variable, whereas the other anthropometric variables were treated as categorical variables. Race/ethnicity included the following categories: Mexican American, Other Hispanic, Non-Hispanic White, Non-Hispanic Black and Other Race (which included Multi-Racial participants). Marital status was categorized into: (I) married/living with partner, (II) widowed/divorced/separated, and (III) never married. The pre-defined NHANES categories for the education level were not modified. The BMI was categorized as follows: (I) obesity (BMI ≥ 30 kg/m^2^), (II) overweight (BMI 25–29.99 kg/m^2^), (III) normal weight (BMI 18.5–24.99 kg/m^2^), and (IV) underweight (BMI ≤ 18.49 kg/m^2^). Following the approach of Giannos et al., we categorized the alcohol intake into 3 groups: (I) low intake (alcohol intake < 15 g per day), (II) moderate intake (15–30 g per day), and (III) high intake (>30 g per day) [42]. Physical activity was dichotomized into 2 groups based on the Physical Activity Guidelines for Americans 2018 [43,44]: (I) low–moderate (less than 150 min of moderate-intensity physical activity per week) and (II) moderate–high (more than 150 min of moderate-intensity physical activity per week). Sleep duration was also dichotomized as follows: (I) less than 7 h per night and (II) more than 7 h per night. Smoking status was identified as non-smoker and current smoker.

### 2.5. Inclusion and Exclusion Criteria

Our study was restricted to male NHANES participants. Only participants who were 20 years or older and who met the following inclusion criteria were included: available anthropometric and demographic data, available nutrient intake data, and available laboratory values. Moreover, we considered only participants who provided information on alcohol intake and smoking, physical activity and sleep duration. Individuals with incomplete or missing data were excluded from this particular analysis.

### 2.6. Statistical Analysis

The statistical analysis was performed with STATA 14 statistical software (StataCorp. 2015. Stata Statistical Software: Release 14. StataCorp LP, College Station, TX, USA). The primary sampling unit variable for the variance estimation and the pseudo-stratum variable as the stratification variable that were provided in the 2015–2016 NHANES dataset were used for this particular analysis.

To check for the normality of the data, we used subpopulation summary statistics and graphical visualizations. Categorical variables were shown as weighted proportions with the standard error in parenthesis. Normally distributed variables were described with their mean and standard error in parenthesis.

The analysis was performed in accordance with the most recent approaches by Heeringa, West and Berglund [45]. Standard errors were estimated using Taylor series linearization to account for the complex NHANES sampling design. To account for differential non-response and/or non-coverage and to adjust for oversampling, we used appropriate sample weights. This allowed for estimated weighted percentages and means that were representative of the noninstitutionalized civilian population.

Considering the most recent data presentation standards for proportions [46], we carefully scanned all the weighted proportions for potential unreliability with the post-estimation Stata command “kg_nchs” [47]. Potentially unreliable proportions that did not meet the NCHS presentation standards were flagged with superscript letters.

Stata’s Rao–Scott test and multivariate linear regression analyses (followed by adjusted Wald tests) were conducted to assess the potential associations between serum testosterone levels and DAL. Multivariate linear regression models were constructed in accordance with the model building techniques of Heeringa, West and Berglund [45]. Furthermore, we investigated the potential differences in the DAL scores between the participants with low testosterone levels and normal testosterone levels, using a cutoff of 300 ng/dL. Based on Sribney’s manual, we estimated the potential correlations between the crude DAL scores and selected steroid hormones [48]. A *p*-value < 0.05 was employed as the cutoff for statistical significance.

## 3. Results

The total sample eligible for analysis comprised *n* = 377 male NHANES participants. The sample characteristics may be obtained from Table 1. Table 1 also displays the sample characteristics stratified by testosterone level (low vs. normal testosterone level). The total sample may be extrapolated to represent 19,433,000 Americans.

The mean age of the sample was 49.50 years. Almost 74% of participants were of Non-Hispanic White origin. Approximately 72% of participants were either married or had a partner. Based on the BMI analysis, 43.48% of participants were overweight and 34.82% of the sample were obese. Only 49.66% of the sample reported more than 150 min of moderate-intensity physical activity per week. Alcohol intake was low in approximately 66% and high in more than 24% of the sample. The entire sample comprised 31.76% smokers and 68.24% non-smokers.

Table 2 displays the nutrient intake in our sample. The mean energy intake in the sample was 2512.86 kcal/d. We observed no significant intergroup differences in the DAL-relevant nutrient intakes when comparing participants with low and with normal testosterone levels.

Table 3 shows the DAL scores in our sample. The mean PRAL_R_ was greater than 0 mEq/d in both groups, indicating an acidifying diet. We observed no significant intergroup differences in the DAL scores when comparing participants with low and with normal testosterone levels.

Table 4 displays the crude DAL scores and their correlations with serum testosterone, sex hormone-binding globulin, and the free androgen index. Significant yet weak correlations were found between the PRAL_R_ and FAI as well as the NEAP_R_ and SHBG.

We performed a multiple regression to predict serum testosterone levels from the PRAL_R_, age, ethnicity/race and body mass index (Table 5, model 1). These variables statistically significantly predicted the serum testosterone levels, F(7,9) = 14.13, *p* < 0.0005, *R*^2^ = 0.17. The PRAL_R_ did not add statistically significantly to the prediction (*p* = 0.411). The association remained insignificant when adjusting for additional variables in models 2 and 3 (Table 5).

## 4. Discussion

The present analysis investigated whether the DAL was associated with serum total testosterone concentrations of men in the U.S.-based National Health and Nutrition Examination Survey (NHANES). Analyzing data from *n* = 377 male NHANES participants, we found no significant associations between the PRAL and NEAP and serum testosterone levels. To the best of our knowledge, we present the first study in the scientific literature to investigate the impact of the DAL on serum testosterone levels in a nationally representative cohort of U.S. males.

Low serum testosterone has been associated with a number of adverse health conditions, for example, obesity, diabetes, an unfavorable lipid profile, reduced bone and muscle mass, and decreased quality of life [49,50,51]. In older men, testosterone insufficiency is associated with an increased risk of death over the following 20 years—a finding that is notably independent of numerous external risk factors and pre-existing health conditions [52]. The number of elderly men will substantially increase in the coming decades and hence their well-being is of general concern for public health [49]. Moreover, testosterone levels may also play an important role in the development of prostate cancer [53], and they have potential implications for the prognosis of prostate cancer patients [54].

In light of these findings, it is of utmost importance to identify environmental and lifestyle factors that could potentially influence testosterone levels. This may apply to both prevention and treatment strategies. More than three decades ago, Adlercreutz postulated that a Western diet elevates the plasma levels of sex hormones and decreases the serum sex hormone-binding globulin concentrations, thereby increasing the bioavailability of these steroids [8].

Our cross-sectional analysis investigated whether the DAL—a novel overall dietary marker focusing on the acidifying/alkalizing character of diets—was associated with testosterone levels. The results, however, suggested no such association.

A high DAL is often the result of a high intake of animal protein and processed grains, accompanied by a low intake of plant foods [18,19]. In contrast, low-PRAL diets are rich in alkalizing foods such as fruits, vegetables, and pulses [33,55]. Such diets are not in line with the common belief that men should adhere to a traditional meat-based diet in order to maintain ideal testosterone levels [56]. Our results revealed no association between the DAL and testosterone levels, suggesting that low testosterone levels are not associated with lower DAL scores (and thus with a higher intake of plant foods and lower intake of animal foods). This is of particular importance for those individuals who wish to lower their DAL in light of the presumable health effects of a more alkaline diet [57,58]. Diet modifications have far-reaching implications [12], and it will be reassuring for men to know that their planned dietary changes toward a lower intake of animal protein and refined grains (which consecutively translates into a lower DAL) may not negatively affect their testosterone levels.

A comparison of our results with other studies remains difficult, since we are, to the best of our knowledge, the first group to assess the relationship between the DAL and testosterone levels in men. Several studies suggested that a lower DAL may favorably affect various medical conditions known to be associated with reduced testosterone (including overweight and type 2 diabetes [59,60,61,62]). As such, one could have expected that a low DAL may beneficially affect testosterone levels. Notably, our results could not confirm this hypothesis. A reservation must be made though, that several studies did not link DAL to adverse health outcomes [63,64], which poses an argument against our overall hypothesis.

The present analysis has several weaknesses but also draws upon a number of strengths. As for the strengths, our study is based on a nationally representative dataset from the NHANES. The modest sample size and the inclusion of important covariates (e.g., physical activity, smoking status, etc.) in our employed multivariate models are an additional asset. We present an innovative hypothesis that has not been examined before. Meanwhile, the weaknesses of our study include the intrinsic limitations of a cross-sectional analysis and the inherent potential for various biases. As explained in detail by Kuchakalla [12], the NHANES does not account for longitudinal changes in diet, serum testosterone levels and unreported comorbidities. Moreover, our analysis did not consider prescribed testosterone supplementation. In addition to that, the testosterone levels were based on a single measure only (as per the NHANES guidelines), whereas some guidelines recommend at least two different measurements to account for intra-individual diurnal serum testosterone variations. We also acknowledge that our study did not include seminal parameters, which may have allowed for additional insights.

Although our results are of interest, additional studies in other populations are warranted to confirm our findings. Prospective studies in particular could help to gain a better understanding of the role of the DAL in sex hormone metabolism. This is of particular importance since a high DAL has been shown to affect glucocorticoid metabolism and secretion in children [65]. Larger studies in different age groups (e.g., adolescents, young males and elderly man) would thus be of great interest.

## 5. Conclusions

The DAL is not associated with testosterone levels in this nationally representative sample of U.S. males. A diet high in alkalizing plant-based foods and low in acidifying foods of animal original may not adversely affect testosterone levels. Additional trials are warranted to confirm our findings. Future studies should ideally employ a prospective randomized controlled design with a sufficiently long study duration, additional DAL markers (e.g., based on 24 h urine samples) as well as seminal fluid parameters.

## Figures and Tables

**Table 1:** Sample characteristics. The total sample comprised *n* = 377 males.

	Total Sample (*n* = 377)	Low Testosterone Level (*n* = 101)	Normal Testosterone Level (*n* = 276)	*p*-Value
**Age (years)**	49.50 (1.53)	51.27 (2.29)	48.88 (01.58)	
**Race/Ethnicity**				0.277 ^b^
Mexican American	6.59% (1.90) *	4.50% (1.64) *	7.33% (2.21) *	
Other Hispanic	5.97% (1.77) *	7.71% (2.42) *	5.35% (1.66) *	
Non-Hispanic White	73.35% (3.73)	76.01% (5.35)	72.41% (3.84)	
Non-Hispanic Black	7.27% (1.21)	6.48% (1.94) *	7.55% (1.45)	
Other Race ^a^	6.82% (1.53)	5.31% (1.90) *	7.36% (1.65)	
**Marital status**				0.650 ^b^
Married/Living with partner	72.33% (2.98)	76.07% (6.34)	71.01% (3.44)	
Widowed/Divorced/Separated	11.36% (2.52)	11.70% (5.18) *	11.23% (2.12)	
Never married	16.31% (2.22)	12.23% (4.68) *	17.75% (3.09)	
**Education Level**				0.219 ^b^
Less than 9th grade	2.01% (0.72)	1.82% (1.08) *	2.07% (0.91)	
9–11th grade	7.76% (1.08)	8.14% (2.61) *	7.62% (1.59)	
High school graduate/GED ^d^	23.62% (3.91)	15.17% (5.80) *	26.60% (4.81)	
Some college or AA degree	34.71% (2.05)	46.96% (7.90) *	30.3% (3.46)	
College graduate or above	31.90% (4.79)	27.91% (5.66)	33.31% (5.08)	
**BMI**				<0.001 ^b^
<18.50	0.36% (0.25)	0% *	0.49% (0.34)	
≥18.50 & <25.00	21.34% (3.34)	7.07% (2.67) *	26.37% (4.33) ^c^	
≥25.00 & <30.00	43.48% (3.68)	36.21% (4.98)	46.05% (4.08)	
≥30	34.82% (4.33)	56.72% (5.67)	27.10% (4.93) ^c^	
**Physical activity**				0.303 ^b^
<150 min per week	50.34% (2.36)	55.17% (5.78)	48.64% (2.34)	
≥150 min per week	49.66% (2.36)	44.83% (5.78) *	51.36% (2.34)	
**Alcohol Intake**				0.076 ^b^
Low	66.21% (3.66)	79.77% (6.91) *	61.43% (3.39) ^c^	
Moderate	9.66% (2.06)	3.91% (2.87) *	11.68% (2.58)	
High	24.13% (3.96)	16.32% (6.37) *	26.89% (4.02)	
**Hours of sleep**				0.243 ^b^
<7 h per day	23.46% (2.26)	17.13% (5.42) *	25.69% (2.57)	
≥7 h per day	76.54% (2.26)	82.87% (5.42) *	74.31% (2.57)	
**Current smoking status**				0.067 ^b^
Smoker	31.76% (3.07)	21.02% (4.98)	35.55% (3.91)	
Non-smoker	68.24% (3.07)	78.98% (4.98)	64.45% (3.91)	

Weighted proportions. Total number of unweighted observations: *n* = 377. Continuous variables shown as the mean (standard error). Categorical variables shown as the weighted proportion (standard error). * Unreliable (weighted) proportions, as per recent NCHS Guidelines. ^a^ Includes Multi-Racial; ^b^ Based on Stata’s design-adjusted Rao–Scott test; ^c^ Indicates significant differences in the weighted proportions; ^d^ Or equivalent.

**Table 2:** Nutrient intake in the selected sample of *n* = 377 males. Total sample (left) and stratified by testosterone level < 300 ng/dL (middle) vs. ≥300 ng/dL (right).

	Total Sample (*n* = 377)	Low Testosterone Level (*n* = 101)	Normal Testosterone Level (*n* = 276)	*p*-Value
Energy intake (kcal/d)	2512.86 (58.82)	2390.29 (92.72)	2556.08 (68.65)	0.137
Protein intake (g/d)	96.20 (1.79)	90.05 (3.95)	98.37 (2.80)	0.171
Phosphorus intake (mg/d)	1619.62 (34.70)	1503.54 (68.46)	1660.55 (45.14)	0.091
Magnesium intake (mg/d)	356.78 (13.33)	320.31 (17.90)	369.64 (19.13)	0.103
Potassium intake (mg/d)	3055.88 (86.83)	2977.89 (160.80)	3083.38 (118.40)	0.639
Calcium intake (mg/d)	1137.75 (38.10)	1097.45 (69.85)	1151.97 (40.47)	0.458

Continuous variables shown as the mean (standard error). A *p*-value < 0.05 indicates a statistically significant difference between the participants with low testosterone levels and normal testosterone levels.

**Table 3:** DAL scores in the selected sample of *n* = 377 males. Values are shown for the total sample (left) and stratified by testosterone levels < 300 ng/dL (middle) vs. ≥300 ng/dL (right).

	Total Sample (*n* = 377)	Low Testosterone Level (*n* = 101)	Normal Testosterone Level (*n* = 276)	*p*-Value
PRAL_R_ (mEq/d)	18.82 (1.82)	14.63 (2.90)	20.30 (2.16)	0.141
NEAP_R_ (mEq/d)	67.45 (1.87)	65.48 (2.89)	68.17 (2.39)	0.507
NEAP_F_ (mEq/d)	60.11 (2.18)	59.04 (3.72)	60.49 (2.18)	0.688

Continuous variables displayed as the mean (standard error). A *p*-value < 0.05 indicates a statistically significant difference between the participants with low testosterone levels and normal testosterone levels.

**Table 4:** Crude dietary acid load scores in mEq/d and their correlations with the serum testosterone (left), sex hormone-binding globulin (middle) and free androgen index (right), based on the entire sample.

	Mean (SE)	Serum Testosterone		Sex Hormone-Binding Globulin		Free Androgen Index	
		*r*	*p*-Value	*r*	*p*-Value	*r*	*p*-Value
PRAL_R_	18.82 (1.82) mEq/d	0.004	0.511	−0.109	0.145	0.151	0.034
NEAP_R_	67.45 (1.87) mEq/d	−0.028	0.680	−0.162	0.042	0.14	0.054
NEAP_F_	60.11 (2.18) mEq/d	0.004	0.965	−0.12	0.125	0.113	0.241

Continuous variables displayed as the mean (standard error).

**Table 5:** Multivariate linear regression models examining the potential associations between testosterone levels, DAL (as assessed via the PRAL_R_) and other covariates.

Independent Variables	β	SE	*p*	β	SE	*p*	β	SE	*p*
	Model I			Model II			Model III		
**PRAL_R_**	0.374	0.44	0.411	0.35	0.33	0.300	0.42	0.43	0.344
**Age**	−1.29	0.71	0.087	−4.72	0.57	<0.001	−4.95	0.48	<0.001
**Ethnicity**									
Mexican American	−12.90	19.92	0.527	−4.26	15.06	0.781	−4.08	16.51	0.808
Other Hispanic	−57.07	20.43	0.014	−13.05	15.62	0.417	−12.70	15.59	0.428
Non-Hispanic Black	45.46	39.81	0.271	32.94	32.39	0.325	38.33	30.52	0.228
Other Race ^a^	0.18	36.18	0.996	−14.24	31.44	0.657	−17.06	29.83	0.576
**Body mass index**	−12.66	2.16	<0.001	−7.90	1.57	<0.001	−8.04	1.50	<0.001
**SBGH**				5.51	0.29	<0.001	5.58	0.29	<0.001
**Smoking status**									
Current smoker							−17.03	18.97	0.384
**Physical activity**									
≥150 min per week							−7.52	15.78	0.653
**Alcohol intake**									
Moderate							−18.98	21.40	0.389
High							−14.62	29.85	0.631
**Energy intake**									
kcal/day							0.0011	0.0126	0.932

^a^ Also includes “Multi-Racial”. Significant regression equations were obtained for all 3 regression models: F(7,9) = 14.13 (model 1); F(8,8) = 42.52 (model 2); and F(13,3) = 59.04, respectively, with a *p*-value < 0.001 for model 1 & 2 and a *p*-value of 0.003 for model 3. The *R*^2^ values were 0.17, 0.49, and 0.50, respectively. The reference categories were as follows: Non-Hispanic White, non-smoker, less than 150 min of moderate physical activity per week, and low alcohol intake.

## Data Availability

The data used in this study are publicly available online (https://wwwn.cdc.gov/nchs/nhanes/Default.aspx; accessed on 2 July 2023).
